# Good things come to those who wait—Decreasing impatience for health gains and losses

**DOI:** 10.1371/journal.pone.0229784

**Published:** 2020-03-03

**Authors:** Stefan A. Lipman, Arthur E. Attema

**Affiliations:** Erasmus School of Health Policy & Management, Erasmus University Rotterdam, Rotterdam, the Netherlands; Shandong University of Science and Technology, CHINA

## Abstract

Historically, time preferences are modelled by assuming constant discounting, which implies a constant level of impatience. The prevailing empirical finding, however, is decreasing impatience (DI), meaning that levels of impatience decrease over time. Theoretically, such changes in impatience are crucial to understand behavior and self-control problems. Very few methods exist to measure DI without being restricted to or confounded by certain assumptions about the discounting function or utility curve. One such measure is the recently introduced DI-index, which has been applied to both monetary and health outcomes. The DI-index quantifies the deviation from constant impatience and is flexible enough to capture both increasing and decreasing impatience. In this study, we apply the DI-index to measure impatience for health outcomes in a reference-dependent framework. That is, we measure impatience for both health gains and health losses compared to a reference-point, in individual and societal settings, using a within-subjects design (n = 98). We allowed for both positive and negative discounting, since negative discounting has been observed for losses (i.e. preferring to incur losses earlier rather than later) in earlier work. To capture changes in time inconsistency when subjects show negative discounting (i.e. patience), we modify the DI-index to a decreasing (im)patience (DIP)-index, which can be applied without loss of generality. As in earlier work, we observe large heterogeneity in time consistency; i.e., a mix of decreasing, increasing and constant (im)patience. Across all DIP-indices elicited, increasing impatience was the modal preference for those satisfying impatience, and decreasing patience for those satisfying patience. No systematic differences were observed between health gains and losses or between societal and individual outcomes. This suggests that for health outcomes both patient and impatient individuals assign more importance to time differences delayed further in the future.

## Introduction

Many things in life will require waiting, as these outcomes will occur later in time. This holds for both good outcomes, such as a coveted holiday, monetary gains or recuperating from surgery, but also for bad outcomes, such as a dreaded presentation, parking fines or side-effects of certain drugs. When making decisions about such outcomes, we have to make intertemporal trade-offs, i.e. trading off later against sooner consequences. For example, we may make choices such as speeding in the car, eating unhealthily, or drinking excessively knowing that their possible negative consequences will only occur later in time. Similarly, we may choose to exercise, do household administration or pay taxes in time, where we may have to wait to receive the positive consequences of such choices.

In economics, discount rates are often used when modelling such preferences at different points in time (i.e. time preferences). These discount rates will be applied to outcomes, typically decreasing their value when they occur later in time. The behavioral impact of such discounting of future outcomes will depend on whether outcomes are gains or losses, i.e. it is sign-dependent. For decisions under risk or uncertainty, it is well known that decisions are different between gains and losses compared to a reference-point [1, 2]. It has also been shown that intertemporal trade-offs are affected by such sign-dependence, where differences in intertemporal choice have been observed between gains and losses [3, 4]. Traditionally, positive discounting is assumed, which implies that the value of positive outcomes will depreciate over time, whilst applying a positive discount rate to losses will reduce their negative impact [5]. As such, this leads to the prediction that we tend to prefer earlier gains to later ones, whilst also preferring to delay losses. Some studies have found negative discounting [e.g. 6, 7, 8], which will lead one to prefer to postpone gains and expedite losses.

However, the sign and size of the discount rate only capture how far one will postpone gains or losses, whilst not deviating from one’s original plan when times passes; whereas, a plethora of literature on self-control problems [e.g. 9, 10, 11] suggests that often we postpone repeatedly (e.g. repeatedly planning to do tax administration next month). Such repeated non-adherence to future plans reflects time inconsistency, which is a crucial component of intertemporal choice. Typically it is modelled by the degree to which discounting remains constant over time, where the prevailing empirical finding for both health and monetary outcomes is decreasing impatience [12–16]. For example, Joe may be willing to wait only 2 months to double a monetary reward otherwise paid out today, but if the original reward was to be paid out in 2 months, he may suddenly be willing to wait 6 months for its doubled counterpart. Whereas the first preference indicates that Joe discounts the future at a certain rate, i.e. has a certain level of impatience, the second preference indicates a change in Joe’s impatience (it decreases). Such decreasing impatience (DI) is also captured by the popular quasi-hyperbolic discounting model [17]. Nonetheless, several studies have found increasing impatience for a considerable proportion of individuals [18–21]. However, it has proven to be difficult (but possible) to empirically disentangle time inconsistency and discount rates, especially as both may be affected by utility curvature [22, 23]. Importantly, non-constant discounting need not imply time inconsistency, as such inconsistencies arise depending on the outcomes and decision problem at hand (see section ‘Time inconsistency’). For example, time inconsistency does not occur when no option to reverse preferences at a later point in time is offered. Nonetheless, for brevity we will use the conventional term time inconsistency to refer to non-constant discounting.

A recent methodological development in the measurement of time inconsistency was proposed by Rohde [18], who introduced the Decreasing Impatience (DI)-Index. The DI-index is an easy-to-elicit summary measure of the degree of deviation from constant discounting. Rohde [18] showed how the DI-index allows distinguishing between levels of impatience, and changes in time impatience, between different periods. She showed how for monetary outcomes the DI-index can capture changes in time inconsistency, without being affected by utility curvature or the level of impatience. The DI-index was also applied to health outcomes, i.e. medical treatments affecting length and quality of life [19].

So far, however, the few studies using the DI-index have mostly focused on gains, whilst it is well known that time preferences differ between gains and losses [3, 4, 22, 24], which holds for health outcomes as well [7, 25]. Surprisingly, the only existing study that used the DI-index separately for gains and losses found no difference across domains, yet this study used only financial outcomes [26]. As such, in this study, we extend earlier work by applying the DI-index to both individual and societal health outcomes as in Attema and Lipman [19], where outcomes span across gains and losses compared to a reference-point. This will allow us to show the extent to which changes in impatience differ between health gains and losses, and if it matters to whom these health outcomes accrue.

This paper is organized as follows. The paper commences with a section containing all theoretical background and notation, and the next section presents details about the experimental approach applied. It continues with a summary of the results of this experimental approach. The final section discusses these findings and concludes the paper.

## Theoretical background

### Notation

We consider timed outcomes of the form (*x*, *t*), where *x* in our experiment denotes a health outcome with time of onset *t*. Preferences ≽ are defined as usual, that is we assume weak-ordered preferences with (strict) preference and indifference denoted by (≻)≽ and ~, respectively. We assume that the decision maker satisfies the discounted utility model, i.e. preferences can be evaluated by:

DU(x,t)=D(t)U(x). (1)

In this general model of intertemporal choice, *D*(⋅) refers to the discount function, which may take different shapes in different discounting models (see Table 1), and *U*(⋅) captures the instantaneous utility of timed outcomes.

**Table 1:** Discounting functions *D*(⋅) for three popular discounting models, with implications for impatience.

Model	Discounting function	Implication for impatience
Constant discounting [5]	*D*(*t*) = 1/(1 + *r*)^*t*^, where *r* reflects the discount rate.	Constant
Quasi-hyperbolic discounting [17]	*D*(*t*) = *β*/(1 + *r*)^*t*^, with 0 < *β* ≤ 1 for *t* > 0 and *D*(*t*) = 1 otherwise, and *r* reflects the per-period discount rate.	Decreasing when *t* = 0 is involved, constant otherwise
Generalized hyperbolic discounting [27]	*D*(*t*) = 1/(1 + *γt*)^*a*/*γ*^, with *a*, *γ* > 0, where *γ* captures differences from constant discounting.	Decreasing throughout

As is summarized in Table 1, popular discounting models assume either that impatience remains constant (i.e. constant or quasi-hyperbolic discounting for *t* ≠ 0) or is decreasing (i.e. generalized or quasi-hyperbolic discounting when this involves *t* = 0). Increasing impatience is not captured by these models, even though this has been consistently observed for both health and monetary outcomes [18–21].

### Time inconsistency

Time inconsistency can arise from both increasing and decreasing impatience, i.e. from non-constant discounting. We assume monotonicity holds, i.e. if *x* ≽ [≻]*y* implies (*x*, *s*) ≽ [≻](*y*, *s*). Impatience holds when (*x*, *s*) ≻ (*x*, *t*) whenever *s* < *t*. Assume we offer an impatient individual two timed outcomes (*x*, *s*) and (*y*, *t*), with *x* ≺ *y*, and find the following indifference (*x*, *s*) ~ (*y*, *t*). Decreasing (increasing) impatience implies that whenever (*x*, *s*) and (*y*, *t*) are delayed by duration *τ* this indifference no longer holds, i.e. (*x*, *s* + *τ*) ≽ (≼) (*y*, *t* + *τ*), whenever *s* < *t*, *τ* > 0. Such preferences can give rise to time inconsistency when this impatient individual is given the opportunity to change their preferences. For example, a decreasingly impatient individual might have the following preferences today: (*x*, *s*) ~ (*y*, *t*) and (*x*, *s* + *τ*) ≺ (*y*, *t* + *τ*). If, after duration *τ* has passed, our respondent is again offered the choice between (*x*, *s*) and (*y*, *t*), she is again indifferent, and might in fact not choose (*y*, *t*).

The most popular measure to capture such non-constant discounting (that could yield time inconsistency) is developed by Prelec [28]. This measure captures decreasing impatience as the Pratt–Arrow degree of convexity of the logarithm of the discount function, i.e. as:

$$P(t)=\frac{{\left[\text{ln}\;D(t)\right]}^{\prime }}{{\left[\text{ln}\;D(t)\right]}^{'}}.$$ (2)

However, this measure of decreasing impatience requires measurement or assumptions about both *D*(∙) and *U*(∙), which complicates its use in practice. Several other measures, which require fewer assumptions, have been developed and capture non-constant impatience more generally, for example the hyperbolic factor [29], the I^3^(t) measure proposed by Rambaud and Fernández [30], and the DI-index [18]. The latter summary measure of changes in time inconsistency, which approximates Prelec’s measure, has several advantages. For example, as opposed to Prelec’s [28] measure of DI, no knowledge about the utility function or assumptions about the discounting function are required [for a recent review of these and other methods, see: 31]. Furthermore, unlike the hyperbolic factor [29], the DI-index does not rely on or assume generalized hyperbolic discounting, and should be able to capture strongly decreasing and increasing impatience. It should be noted here that the recent I^3^(t) index [30], of which we were unaware when conducting our study, also has these properties, although it does require information about the shape of the discounting function.

### DI-index

To derive the DI-index, Rohde [18] considers combinations of two outcomes (*x*, *s*) and (*y*, *t*) and assumes monotonicity and impatience. She defines impatience for both gains and losses: as *x* ≺ [≻]0 implying for all *s* < *t*: (*x*, *s*) ≺ [≻](*x*, *t*), i.e. one prefers to delay bad outcomes and speed up good outcomes, which are defined relative to reference outcome 0. The DI-index is then obtained by means of two indifferences: (*x*, *s*) ~ (*y*, *t*) and (*x*, *s* + *σ*) ~ (*y*, *t* + *τ*) with *x*, *y* ≁ 0, *s* < *t*, *σ* > 0, and calculated by:

$$DI-index=\frac{\tau -\sigma }{\sigma (t-s)}.$$ (3)

Whenever the DI-index is positive, zero or negative, this corresponds to decreasing, constant or increasing impatience, respectively.

In other words, the DI-index can be obtained by eliciting two indifferences for two non-zero timed outcomes. First, one elicits the delay required to yield indifference between outcomes *x* and *y*, obtained at times *s* and *t*, respectively. Second, outcome *x* is delayed further by *σ*, and indifference is elicited which gives the additional delay *τ* required to yield indifference.

### Reference-dependent DI-index and negative time preference (patience)

Thus far, the DI-index has been applied to both monetary [18] and health outcomes [19]. In both cases, these outcomes referred to gains, where in the former study the outcome 0 was implicitly used as reference-point, whilst the latter study involved gains in quality of life from a reference-point which consisted of a reduced health state. In this study, we extend earlier work by eliciting DI-indices for both health gains and health losses. Although the notation used by Rohde [18] allows for outcomes to be losses, several changes to her approach are required to consistently measure DI-indices for health losses.

First, we will assume that outcomes *x* are evaluated as compared to a reference-point *x*_0_. We define this reference-point (RP) as a point of comparison, which can be different during different parts of the experiment. Given that no plausible theory of RP selection is available [32], we let the RP depend on framing of the decision context and assume that the decision maker adopts the RP described. Gains and losses are defined as timed outcomes preferred to RP, i.e. any (*x*, *t*) ≻ (*x*_0_, *t*) is a gain and any (*x*, *t*) ≺ (*x*_0_, *t*) is a loss, where *x*_0_ is the RP.

Second, it is well known that some individuals have negative time preference, especially for losses (e.g. 8). Such preferences capture that instead of preferring to delay losses, one may be inclined to incur them earlier, to ‘get it over with’. Similarly, one may prefer to delay a gain, to ‘save the best for last’ [4, 6]. When these preferences are to be captured by the discount function, the discount rate (e.g. *r* in constant and quasi-hyperbolic discounting) is taken to be negative. Such negative discounting will lead one to prefer to delay gains and dislike delays of losses. We extend the framework used by Rohde [18] to include such preferences, by extending the assumption of impatience throughout. Instead, we modify this assumption to allow for either patience or impatience, where this is defined sign-dependently. For example, decision makers can be impatient for gains and patient for losses. Whenever *x* ≺ [≻]*x*_0_ implies (*x*, *s*) ≺ [≻](*x*, *t*) for all *s* < *t*, *impatience* holds, and whenever *x* ≺ [≻]*x*_0_ implies (*x*, *t*) ≺ [≻](*x*, *s*) for all *s* < *t*, *patience* holds.

This assumption implies some changes in the interpretation and elicitation of the DI-index. The definition by Rohde [18] implies that the DI-index captures changes in the absolute rate at which our willingness to wait increases or decreases (assuming impatience). In our study, we adapt the derivation of the DI-index such that it can reflect the extent of changes in willingness to wait in either patience or impatience, and thus it reflects to what extent any discount rate decreases or increases, regardless of its initial sign. As in Rohde [18], this index can be obtained by means of two indifferences, where the derivation differs when decision makers are patient or impatient. For simplicity, we assume either *x*_0_ ≻ *x* ≻*y* (losses), or *y* ≻ *x* ≻*x*_0_ (gains), i.e. both outcomes are either gains or losses. As in Rohde [18], whenever impatience holds and *s* < *t*, *σ* > 0, the indifferences (*x*, *s*) ~ (*y*, *t*) and (*x*, *s* + *σ*) ~ (*y*, *t* + *τ*) allow derivation of the DI index by Eq 3. Whenever patience holds and *s* < *t*, *σ* > 0, (*y*, *s*) ~ (*x*, *t*) and (*y*, *s* + *σ*) ~ (*x*, *t* + *τ*), the decreasing patience (DP)-index is calculated by:

$$DP-index=\frac{\tau -\sigma }{\sigma (t-s)}.$$ (4)

This derivation of the DP-index ensures that whenever the DP-index is positive, zero or negative, this corresponds to increasing, constant or decreasing patience, respectively.

As such, both the DI- and DP-index reflect changes in the absolute rate at which our willingness to wait increases or decreases. This holds regardless of whether discounting is negative or positive or if outcomes are gains or losses (see Table 2 for examples). Hence, we will compile DI and DP-indices and refer to this as the DIP-index, a summary measure of time consistency. A single combined definition for the DIP-index can be found in Box 1.

**Table 2:** Derivation and interpretation of DI and DP indices for gains and losses.

**Gains (*y* ≻ *x* ≻ *x***_**0**_**)**								
**Patient (*x*, *s*) ≺ (*x*, *t*)**	**(*y*, *s*) ~ (*x*, *t*)**	**(*y*, *s* + *σ*) ~ (*x*, *t* + *τ*)**	***s***	***t***	***σ***	***τ***	**DP-index**	**Interpretation**
Increasing	(*y*, 0) ~ (*x*, 5)	(*y*, 4) ~ (*x*, 16)	0	5	4	8	0.2	Prefers waiting for gains, and *willingness to wait increases by delays*
Constant	(*y*, 0) ~ (*x*, 5)	(*y*, 4) ~ (*x*, 12)	0	5	4	4	0	Prefers waiting for gains, and *willingness to wait is constant*
Decreasing	(*y*, 0) ~ (*x*, 5)	(*y*, 4) ~ (*x*, 10)	0	5	4	2	-0.1	Prefers waiting for gains, and *willingness to wait decreases by delays*
**Impatient (*x*, *s*) ≻ (*x*, *t*)**	**(*x*, *s*) ~ (*y*, *t*)**	**(*x*, *s* + *σ*) ~ (*y*, *t* + *τ*)**	***s***	***t***	***σ***	***τ***	**DI-index**	**Interpretation**
Decreasing	(*x*, 0) ~ (*y*, 5)	(*x*, 4) ~ (*y*, 16)	0	5	4	8	0.2	Dislikes waiting for gains, and *willingness to wait increases by delay*
Constant	(*x*, 0) ~ (*y*, 5)	(*x*, 4) ~ (*y*, 12)	0	5	4	4	0	Dislikes waiting for gains, and *willingness to wait is constant*
Increasing	(*x*, 0) ~ (*y*, 5)	(*x*, 4) ~ (*y*, 10)	0	5	4	2	-0.1	Dislikes waiting for gains, and *willingness to wait decreases by delays*
**Losses (*x***_**0**_ **≻ *x* ≻ *y*)**								
**Patient (*x*, *s*) ≻ (*x*, *t*)**	**(*y*, *s*) ~ (*x*, *t*)**	**(*y*, *s* + *σ*) ~ (*x*, *t* + *τ*)**	***s***	***t***	***σ***	***τ***	**DP-index**	**Interpretation**
Increasing	(*y*, 0) ~ (*x*, 5)	(*y*, 4) ~ (*x*, 16)	0	5	4	8	0.2	Dislikes waiting for losses, and *willingness to wait increases by delays*
Constant	(*y*, 0) ~ (*x*, 5)	(*y*, 4) ~ (*x*, 12)	0	5	4	4	0	Dislikes waiting for losses, and *willingness to wait is constant*
Decreasing	(*y*, 0) ~ (*x*, 5)	(*y*, 4) ~ (*x*, 10)	0	5	4	2	-0.1	Dislikes waiting for losses, and *willingness to wait decreases by delays*
**Impatient (*x*, *s*) ≺ (*x*, *t*)**	**(*x*, *s*) ~ (*y*, *t*)**	**(*x*, *s* + *σ*) ~ (*y*, *t* + *τ*)**	***s***	***t***	***σ***	***τ***	**DI-index**	**Interpretation**
Decreasing	(*x*, 0) ~ (*y*, 5)	(*x*, 4) ~ (*y*, 16)	0	5	4	8	0.2	Prefers waiting for losses, and *willingness to wait increases by delays*
Constant	(*x*, 0) ~ (*y*, 5)	(*x*, 4) ~ (*y*, 12)	0	5	4	4	0	Prefers waiting for gains, and *willingness to wait is constant*
Increasing	(*x*, 0) ~ (*y*, 5)	(*x*, 4) ~ (*y*, 10)	0	5	4	2	-0.1	Prefers waiting for gains, and *willingness to wait decreases by delays*

**Box 1. Definition of DIP-index**

If *x* ≻ *x*_0_ and (*x*, *s*) ≻ (*x*, *t*) for all *s* < *t*, ***or***

 *x* ≺ *x*_0_ and (*x*, *s*) ≺ (*x*, *t*) for all *s* < *t*,

the DIP-index is obtained by means of the following two indifferences:

(*x*, *s*) ~ (*y*, *t*) and (*x*, *s* + *σ*) ~ (*y*, *t* + *τ*) with *s* < *t*, *σ* > 0.

If *x* ≻ *x*_0_ and (*x*, *s*) ≺ (*x*, *t*) for all *s* < *t*, ***or***

 *x* ≺ *x*_0_ and (*x*, *s*) ≻ (*x*, *t*) for all *s* < *t*,

the DIP-index is obtained by means of the following two indifferences:

(*y*, *s*) ~ (*x*, *t*) and (*y*, *s* + *σ*) ~ (*x*, *t* + *τ*) with *s* < *t*, *σ* > 0.

The DIP-index is then calculated by: $DIP-index=\frac{\tau -\sigma }{\sigma (t-s)}$.

Whenever the DIP-index is positive, zero or negative, this corresponds to increasing willingness to wait, constant willingness to wait or decreasing willingness to wait, respectively.

## Experiment

### Sample and design

An experiment was conducted at the Erasmus Behavioural Lab, where subjects were recruited via the Erasmus Research Participation system. A total of 98 Business Administration students took part in this study, and were rewarded course credits after participating. This study used a 2 × 2 within-subjects design with the following conditions: context (individual vs. societal) and framing (gains vs. losses). That is, DI-indices were elicited for four conditions: individual gains, individual losses, societal gain and societal losses. For each condition, two DI-indices were elicited, by using the following stimuli durations: 0 months, 4 months and 8 months. The experiment was completely randomized, meaning that the order of conditions was random, and within each block the stimuli-durations were randomized as well. This experiment was conducted using Qualtrics Survey Software.

### Health state descriptions

Throughout, we used the EQ-5D-5L classification system [33] to describe health states, both for the reference-point and for gains and losses compared to this health state. This classification system uses five dimensions to describe health-related quality of life: mobility, self-care, usual activities, pain and anxiety/depression. The five-level version of this classification system used five categories to indicate problems faced on these five dimensions of quality of life, which range from ‘no problems’ to ‘extreme problems/unable to…’. Typically, 5-digit codes are used to abbreviate the health states, where each number refers to the level of problems faced on the domains. For example, 11111 refers to no problems on any dimension, and 21231 indicates some problems with mobility, no problems with self-care, some problems with usual activities, moderate pain and no anxiety or depression. We will use this shorter notation for health states in the remainder of the paper.

### Procedure

To distinguish between gains and losses, two RPs were used (see S1 File). For gain framings, we instructed subjects to imagine that they (individual) or a group of 50 students (societal) suffered from a chronic back pain. The health status (state Z) associated with chronic back pain was 31331, i.e. a state with moderate problems with mobility and usual activities and moderate pain. Subjects were offered choices between Treatment A and Treatment B, which would yield temporary improvements in this health status (i.e. gains). For loss framings, subjects had to consider that they (individual) or a group of 50 students (societal) had no problems on any dimensions, i.e. state 11111. As a result of contracting a disease, subjects were offered a choice between Treatment A and B, which as a result of side-effects would yield a temporary reduction in health status. Treatment was necessary; without treatment the disease would be fatal. Importantly, the assumption that we will maintain throughout is that subjects adopt the RP described to them in these hypothetical scenarios, i.e. for gain framings *x*_0_ = 31331, and for loss framings *x*_0_ = 11111. Under this assumption, and assuming monotonicity, any improvement in health status *x* in gain framings should satisfy (*x*, *t*) ≻ (*x*_0_, *t*) and decreases in health status *x* should satisfy (*x*, *t*) ≺ (*x*_0_, *t*), i.e. Treatment A and B are both gains in the gain framing, and both losses in the loss framing. Throughout, the duration of gains and losses in health status realized via Treatment A or B was always exactly one month, which would start directly after treatment had commenced. Directly after this period of one month, subjects would return to *x*_0_, that is chronic back pain would return for gain framings, or health status would return to perfect health in loss framings.

The indifferences required to determine the DI or DP-index were all elicited by means of choice list methodology (see S1 and S2 Figs for an example). Each choice list looked similar, with Treatment A on the left-hand side having a constant delay before treatment (i.e. *s* = 0, 4, 8 months), whilst Treatment B on the right-hand side featured a monotonically increasing delay before treatment. Crucial to our approach here, was determining if subjects discounted positively or negatively (i.e. discounting sign), as this determined which Treatment yielded the larger gain/loss. We determined discounting sign by describing a gain (in gain framings) or a loss (in loss framings), and asking subjects if they would prefer to receive this outcome earlier or later when this outcome would occur in 8 months. As described, preferring to receive gains earlier and losses later implies positive discounting, whilst the reversed preferences imply negative discounting. The outcomes used for our choice list methodology can be found in Table 3.

**Table 3:** Outcomes used in choice list methodology in EQ-5D-5L notation.

Individual	Treatment A (*x*)	Treatment B (*y*)
Gains (*x*_0_ = 31331)	31131 for you	11111 for you
Losses (*x*_0_ = 11111)	21111 for you	31311 for you
		
Societal	Treatment A (*x*)	Treatment B (*y*)
Gains (*x*_0_ = 31331)	21111 for 40 students	21111 for 50 students
Losses (*x*_0_ = 11111)	11121 for 40 students	11121 for 50 students

Because our experiment was computerized, several methodological improvements were possible as opposed to earlier work on DI-indices using pen-and-paper questionnaires to elicit choice list indifferences [18, 19]. Typically, these earlier studies were not able to calculate DI-indices for a considerable part of the sample, which we aimed to remedy with the following changes. First, before starting the choice list elicitation, subjects had to pass a total of four comprehension checks, aimed at testing if they were aware of: a) which treatment provided the better outcome, b) the duration of the gains or losses in health, c) what happened after this duration (i.e. return to the RP), and d) if they understood the descriptions of outcomes. Second, choice lists did not allow violations of monotonicity (i.e. subjects preferring to receive a small health gain after 3 months to a larger gain also after 3 months); if subjects attempted to submit a dominated response they received a warning message explaining why this was considered an error and asked to reconsider. Third, by using a two-staged elicitation approach, i.e. a first choice list eliciting indifference in years and a follow-up choice list which elicited indifference in months, we were able to elicit indifference on a wide range (between 0–10 years), whilst also being relatively precise (in months). If a subject did not switch within these 10 years, choice lists were continued between 10 and 40 years in 2.5-year increments. Only if no switching occurred within these bounds were we not able to calculate DI- or DP-indices. Finally, choice list completion was facilitated for subjects by automatically filling in all dominated options above or below their selection. For example, if a subject with positive discounting indicated preferring Treatment A (gain) in 12 months, impatience implies that they should also prefer Treatment A between now and 11 months, and hence these options were automatically selected. Subjects could freely change this at any point. Importantly, this process was programmed to prevent multiple switching, which allowed us to precisely determine indifferences without the need to exclude subjects.

### Measures

Subjects completed a total of 12 choice lists (3 per condition), where for each choice list Treatment A had a constant duration of *s* = 0, 4, 8 months. As is described by Rohde [18], these 12 indifferences allow the derivation of a total of 8 DI- or DP-indices (2 per condition). In our experiment we obtain *s*, *t*, *τ* and *σ* (required for solving Eq 3) by the following process (applied with *i* = 0 and *i* = 1): we set *s*_*i*_ with *s*_0_ = 0 months, *s*_1_ = 4 months, *s*_2_ = 8 months. This will give *s* as used in Eq 3, by taking *s* = *s*_*i*_. The elicited indifference, which will differ between patient and impatient respondents gives *t* = *t*_*i*_. For impatient respondents, we elicit (*s*_*i*_: *x*) ~ (*t*_*i*_: *y*), whereas for patient respondents we elicit (*s*_*i*_: *y*) ~ (*t*_*i*_: *x*). Next, we obtain *σ*, which corresponds here to *σ* = *s*_*i*+1_ − *s*_*i*_, and elicit the indifference (*s*_*i*+1_: *x*) ~ (*t*_*i*+1_: *y*) or (*s*_*i*+1_: *y*) ~ (*t*_*i*+1_: *x*) for impatient or patient respondents, respectively. We proceed by finding *τ* by determining *t*_*i*+1_ − *t*_*i*_, allowing us to calculate DI- or DP-indices. This process is completed both for gains (with *x*_0_ ≻ *x* ≻ *y*) and losses (with *y* ≻ *x* ≻ *x*_0_). We will apply condition- and duration-specific notation for DI- and DP-indices, meaning that DI-IG-04 indicates a decreasing impatience index for individual gains, calculated with *s*_0_ = 0 and *s*_1_ = 4. Similarly, DP-SL-48 indicates a decreasing patience index calculated for societal losses, calculated with *s*_1_ = 4 and *s*_2_ = 8. Whenever we compile DI- and DP-indices we will write DIP, e.g. the compiled summary measure for societal losses with *s*_0_ = 0 and *s*_1_ = 4 is denoted DIP-SL-04. Finally, several demographics were measured after the choice lists were completed, that is: age, sex, height, and weight. Both current health and life satisfaction were measured with a visual analogue scale ranging from 0–100.

## Results

**Fig 1:**
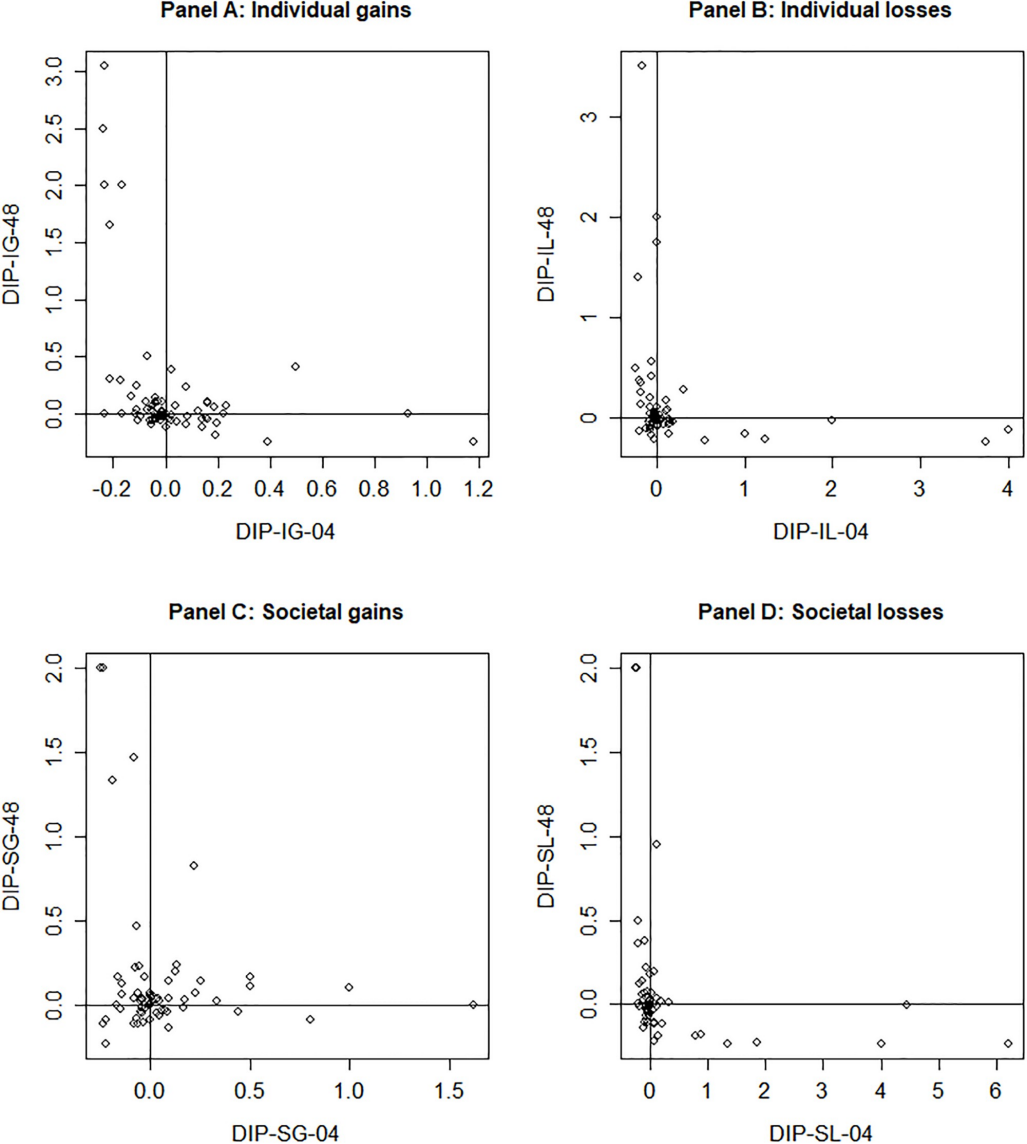
Distribution of DIP-indices per condition.

Even though many methodological improvements were in place to reduce the proportion of subjects for whom no DIP-indices could be calculated, a total of 20% of DIP-indices could not be calculated. Of these missing values, 95% was the result of subjects always choosing the (im)patient option, i.e. always preferring Treatment A and not switching in the choice list. The other 5% of missing values resulted of subjects violating (im)patience, i.e. subjects that were classified as either positive or negative discounters who show indifferences that violate this assumption. In total, we have incomplete data for 52 out of 98 subjects. A second possible violation was transitivity, for example when a respondent has the following preferences: (x,0)~(y,7) and (x,4)~(y,6). Such violations occurred frequently, i.e. from as low as 10% of complete responses for DIP-IG-04 to as much as 49% of responses for DIP-SG-04. In fact, only for 8 respondents all DIP-indices could be calculated based on indifferences for which transitivity holds. Due to this large number of violations, we chose a lenient exclusion rule, i.e. only excluding subjects for whom no DIP-indices could be calculated in any condition. That means our data includes DIP-indices based on indifferences that violate transitivity, and for respondents for whom no DIP-index could be calculated in some but not all conditions. We checked the effects of this lenient exclusion rule, by repeating our analysis with two strict exclusion rules. First, we repeated our analysis with the 46 subjects with complete data. Second, we repeated our analysis on all complete data that satisfied transitivity. In both cases, we reach similar conclusions. Hence, the analysis reported below apply the lenient rule, which required us to exclude just 2 subjects (i.e. *n =* 96). Results for the sample under both strict exclusion rules are available on request. The distribution of DIP-indices can be found in Fig 1. Given that all DIP-indices were not normally distributed (Shapiro-Wilk tests, *p’s* <0.001), we will use non-parametric analyses and report medians with interquartile ranges.

Table 4 shows summary statistics for each condition. As expected, for gains we observed positive discounting for the majority of our sample (85% for individual, 90% for societal gains), whilst for losses we find negative discounting for the majority of the sample (79% for individual, 75% for societal). These proportions were all significantly different than would be expected if positive and negative discounting were independently distributed (Chi-squared, *p’s* <0.001). We find no evidence for an immediacy effect (i.e. where willingness to wait changes substantially for durations involving the present, and less so when the present is not involved). DIP-indices are not significantly higher (one-tailed paired Wilcoxon’s tests, all *p’s* > 0.07) for durations involving the present (e.g. DIP-IG-04 vs DIP-IG-48). Quasi-hyperbolic discounting typically only implies impatience. The analysis reported here captures the degree to which an ‘immediacy effect’ exists (i.e., it reports the degree to which DIP-indices involving the present (s = 0, e.g. DIP-IG-04) are larger than those elicited with s = 4 (e.g. DIP-IG-48). For this analysis to test this immediacy effect for patient individuals, we must assume that quasi-hyperbolic discounting holds (as in Table 1), with β > = 1 and r < 0. These conclusions hold when we combine all DIP-indices involving the present (i.e. DIP-04 combined for all conditions compared to DIP-48 combined for all conditions). Further pair-wise comparisons within context (e.g. societal gains vs. societal losses) and within framing (e.g. societal gains vs. individual gains) suggested that no significant differences existed between the elicited DIP-indices (paired Wilcoxon’s tests, all *p’s* > 0.39), with the exception of a significant difference (paired Wilcoxon tests, *p* = 0.001) between DIP-indices elicited for individual gains and losses (DIP-IG-48 and DIP-IL-48). However, for all conditions we were not able to reject the null-hypothesis that DI or DP-indices (or combined DIP-indices) were equal to 0 (Wilcoxon’s tests, all *p’s* > 0.08).

**Table 4:** Median DIP-indices with first (Q1) and third (Q3) quartile, split for positive and negative discounting.

	Positive discounting					Negative discounting			
	Median	*Q1*	*Q3*	*n*		Median	*Q1*	*Q3*	*n*
**DI-IG-04**	-0.02	-0.06	0.02	73	**DP-IG-04**	0.00	-0.04	0.09	9
**DI-IG-48**	0.00	-0.03	0.10	72	**DP-IG-48**	-0.02	-0.06	0.00	8
**DI-IL-04**	0.00	-0.03	0.07	11	**DP-IL-04**	0.00	-0.05	0.04	68
**DI-IL-48**	-0.01	0.06	0.00	11	**DP-IL-48**	0.00	-0.05	0.10	66
**DI-SG-04**	0.00	-0.06	0.05	74	**DP-SG-04**	0.08	0.05	0.86	4
**DI-SG-48**	0.01	-0.02	0.07	74	**DP-SG-48**	-0.09	-0.16	-0.02	4
**DI-SL-04**	-0.01	-0.06	0.05	18	**DP-SL-04**	0.00	-0.07	0.07	59
**DI-SL-48**	-0.01	-0.04	-0.01	15	**DP-SL-48**	0.00	-0.07	0.04	58

Table 5 shows how subjects were classified, i.e. if they showed increasing, constant or decreasing (im)patience across gains and losses (using the classification rules below Eqs 3 and 4). For both individual and societal contexts, the modal preference was to report increasing impatience and decreasing patience. Both cases signify reduced willingness to wait when outcomes are delayed (i.e. DIP-index < 0). However, a large minority of subjects also reports increased willingness to wait, i.e. decreasing impatience or increasing patience. We compared the distributions within each condition by means of Chi-squared tests. These analyses show that when comparing separately for those who discount positively (i.e. DI-indices) and negatively (DP-indices), or when compiling both indices (DIP-indices), this distribution was not independent (all p’s <0.03). The only exception was the distribution of DP-indices for societal gains (Chi-squared test, p = 0.14). This suggests that for almost all conditions and discounting signs, constant (im)patience was less likely to occur. Next, we drop those respondents with constant impatience, to compare if those with non-constant (im)patience were more likely to have increasing or decreasing willingness to wait. These analyses indicated that if (im)patience was non-constant, it was more likely to reflect decreasing willingness to wait (i.e. increasing impatience or decreasing patience, DIP-index > 0), as these distributions were not independent for each type of discounting and condition (Chi-squared test, all p’s < 0.04), except for DP-indices for individual and societal gains (Chi-squared test, p’s >0.32). When comparing the distributions of DIP-indices per condition, we again find significantly more decreasing willingness to wait (Chi-squared test, all p’s <0.02), except for societal gains (Chi-squared test, p = 0.57).

**Table 5:** Frequency distributions of changes in the degree of (im)patience per condition and separated for patient and impatient individuals.

	**Individual**						
	**Positive discounting (DI-indices)**			**Negative discounting (DP-indices)**			
Implication	Gains	Losses	Total	Gains	Losses	Total	Implication
Decreasing impatience (DI-index > 0)	51	6	**57**	6	46	**52**	Increasing patience (DP-index > 0)
Constant impatience (DI-index = 0)	18	0	**18**	1	13	**14**	Constant patience (DP-index = 0)
Increasing impatience (DI-index < 0)	76	16	**92**	10	75	**85**	Decreasing patience (DP-index < 0)
Total	**145**	**22**		**17**	**134**		
	**Societal**						
	**Positive discounting**			**Negative discounting**			
	Gains	Losses	Total	Gains	Losses	Total	
Decreasing impatience (DI-index > 0)	67	9	**76**	4	38	**45**	Increasing patience (DP-index > 0)
Constant impatience (DI-index = 0)	7	0	**7**	0	21	**21**	Constant patience (DP-index = 0)
Increasing impatience (DI-index < 0)	74	24	**96**	4	58	**62**	Decreasing patience (DP-index < 0)
Total	**148**	**33**		**8**	**117**		

This table compiles the two DI-indices derived for each condition, e.g. DI-04-IG and DI-48-IG

Finally, to qualify our findings, we analyzed subjects’ DIP-indices by means of mixed effects regressions (using the R package lmertest). To this end, we compiled all observations and ran a linear mixed effects model with subject random effects. First, we included fixed effects for: a) context (individual vs. societal), b) framing (gains vs. losses), c) condition (interaction term between context and framing), d) discounting sign (positive vs. negative), and e) measurement (0–4 vs 4–8). Second, we included fixed effects for f) gender (male vs. female), g) BMI, and h) age, to see if any of the effects were reduced or increased by controlling for demographics. In both models, none of the fixed effects was significant (*p’s* > 0.15). This confirms the finding that that no significant or systematic difference existed between the DIP-elicitations per condition or between respondents with different demographic characteristics other than differences in discounting.

## Discussion

In this study, the recently introduced DI-index [18] was applied for the first time to measure time inconsistency for both health gains and losses within the same study. Furthermore, given that negative discounting was frequently observed for losses (e.g. 6, 8), the procedure to elicit the DI-index was modified to enable it to detect changes in the level of negative discounting (i.e. patience). This modification yielded the DP-index, and combining both yielded a DIP-index, which can reflect both changes in impatience and patience. In this study, we were able to obtain such measures of time consistency for both individual and societal health gains and losses, which are not affected by utility curvature or the level of discounting. We find increasing impatience and decreasing impatience to be the most prevalent. This implies that regardless of subjects’ tendency to prefer to wait for or speed up the reception of certain outcome, their willingness to wait decreases by delays. A large majority of our subjects also satisfied decreasing impatience and increasing patience (i.e. increased willingness to wait), and although all DIP-indices were not significantly different from 0, few respondents satisfy the constant (im)patience traditionally assumed in discounted utility models with a constant discount rate. Although many methodological improvements were realized as opposed to earlier studies measuring DI-indices, we find no systematic differences between individual and societal contexts, or between health gains and losses.

Our findings for individual gains are comparable to those of Rohde [18], who elicited the DI-index for monetary outcomes. Bleichrodt and colleagues [21] reported similar results for individual health and monetary outcomes, although they did not consider losses and used the hyperbolic factor, which is only able to capture moderate amounts of decreasing and increasing impatience. Attema and Lipman [19] observed similar results as well for the DI-index for individual and societal health gains, using a pencil-and-paper design. However, these three studies all found a sizable minority of increasing impatience, whilst we report a majority of increasing impatience. Finally, contrary to Attema and Lipman [19], we do not find significant differences between the DIP-indices for individual and societal outcomes. Recently, Shiba and Shimizu [26] compared time inconsistency for gains and losses in the monetary domain and found similar results as we, with a positive correlation between gains and losses, and a substantial amount of increasing impatience in both domains. The results of our study confirm that the degree of time inconsistency is related between gains and losses in health, even when discount rates are not. A possible explanation for this pattern is a nonlinear perception of time [34], which may be independent of the sign of the outcome [35].

Whereas this study makes a number of methodological improvements compared to previous work, its findings should still be interpreted in light of several limitations. First, even though increasing the maximum delay and prohibiting multiple switching appeared to have decreased the amount of necessary exclusions, we still could not calculate DIP-indices for 20% of our sample, which in the vast majority of cases was the result of subjects always preferring the option with the better outcome. These preferences may imply that subjects do not discount health, or at such a low rate that it is not captured by durations of 40 years. The current elicitation procedure of the DIP-index relies on indifferences, and when monotonicity holds, these will not occur without discounting. However, if one satisfies quasi-hyperbolic discounting with a zero discount rate, one could still show decreasing impatience, but this cannot be captured by the elicitation of the DI-index used here. How to calculate the DIP-index when neither impatience nor patience holds is an interesting venue for future research. Second, the lack of differences between the conditions used in this study appears puzzling in light of the study design. Although the computerized design allowed us to improve on internal validity, none of the differences observed in earlier work [19], such as between individual and societal outcomes, was observed in this study. This leads us to question how much of the observed findings are generated by noise or imprecision in preferences [as argued for risk: 36]. Given that subjects received no financial incentives for completing the study, and the within-subjects design may have been perceived as repetitive, some noise or imprecision in preferences could be expected. Hence, if the methodology used to measure DIP-indices here is much more precise than the preferences in question, the estimates may reflect this imprecision to a larger extent than systematic heterogeneity between the different conditions used here. Another explanation for the lack of differences observed here is that earlier work, by excluding inconsistent subjects, has yielded selection bias. It is crucial that future research further explores the robustness and validity of the DI-index as a method to quantify time inconsistency (which could be an exploration of the validity of the DI-index for non-separable discounting models). Importantly, the current studies using DI have all used Dutch student samples. Hence, future research should strive to include a non-student sample to test the external validity of the current and previous findings, for example by applying the DI-index in the general public or in applied work outside the Netherlands (e.g. in non-OECD countries). Even though the method is simple to administer (needing only two indifferences), the combined conclusions of this study for health outcomes and earlier studies that found no relation between DI-indices and various health behaviors [18, 19] question the applicability of measuring time inconsistency by the DI-index, at least for health outcomes.

In conclusion, this study has several implications. First, the DI-index can be extended to measure time inconsistency when patience holds instead of impatience, without loss of generality. Second, constant (im)patience, as assumed in discounted utility models with constant or quasi-hyperbolic discounting only holds for very few subjects. This implies that discounting models that can accommodate changes in (im)patience are required. Third, as documented in earlier work, increasing impatience was observed frequently, in fact it was the modal observation in this study. Patient respondents, however, were more likely to show decreasing patience. Seeing as many individuals were patient for losses, our findings stress the need for general models of intertemporal choice for gains and losses that allow both increasing and decreasing (im)patience. Fourth, the results in this study suggest that once the measurement of decreasing (im)patience allows negative discounting, no differences in time inconsistency are observed between health gains and losses or between individual and societal health outcomes. Future work should explore if this lack of evidence holds for different samples and elicitation procedures.

## Supporting information

S1 File Instructions and choice lists used in experiment. (DOCX)

S1 Fig Example of first stage individual choice list, with s = 0. (TIF)

S2 Fig Example of second stage individual choice list, with s = 0, if first stage yields 2 years. (TIF)

S1 Data Research data. (CSV)
