# Supplementary material for: Good things come to those who wait—Decreasing impatience for health gains and losses
Source: PLoS One. 2020 Mar 3;15(3):e0229784. doi: 10.1371/journal.pone.0229784 (PMC7053719; doi:10.1371/journal.pone.0229784)
Supplement: S1 File — (DOCX) [file pone.0229784.s001.docx]

# Supporting Information

**S1 File: Instructions and choice lists used in experiment**

This appendix reprints the instructions used in the experiment reported on in section 3. Figures A1 to A2 show example screenshots of the choice lists used for individual conditions. Societal choice lists were identical, except that they also listed the students who would receive treatment (i.e. 40 or 50). All instructions below are depicted assuming positive discounting, where for subjects satisfying negative discounting we reversed the order of the outcomes (see Section 2).

S1.1. General Instructions – practice choice list with monetary amounts included

In the following sections you will be asked to choose between several treatment options under different scenarios. The questions will differ in the starting time of the treatments, the scenario that is described and the beneficiaries of the treatments. Throughout this section of this survey, we will use choice lists, which we introduce in some detail to you before moving on to the questionnaire. In this example, we will use a choice list that features monetary outcomes, for clarity purposes.

Consider the example below: in the first row, you will choose option B, because it offers more money and both rewards are paid out at the same time. As you move down the list, option B becomes less attractive because you have to wait longer before the reward will be paid out. In some row, you may probably choose option A. If so, you will also choose option A in all rows below that one, because in these rows option B is even less attractive (you have to wait even longer). Similarly, if you choose option B in a given row, you will also choose option B in all rows above that one, because in these option B is more attractive. The computer will do this for you automatically.

S1.2. Example Instructions – Individual Gains

In this section we ask you to imagine that you have chronic back pain, which is characterized by the following chronic health state (Health State Z):
 -You have **moderate** problems in walking about.

-You have **no** problems in washing or dressing yourself.

-You have **moderate** problems doing your usual activities (e.g. work, study, housework, family or leisure activities).

-You have **moderate** pain or discomfort.

-You are **not** anxious or depressed.

In this case there are two treatments that give you a temporary relief of your symptoms:

**- Treatment A** completely takes away the pain during one month. It does not improve your walking and usual activity problems (Health State X).

**- Treatment B** also completely takes away the pain during one month. In addition, it allows you to walk with only slight problems and to perform your usual activities with *no problems*(Health State Y).

The effects of the treatments start immediately at the beginning of the treatment and last for exactly one month. After this the students return to their usual health state Z.

In the first row, you will choose option B, because it offers a larger improvement in health and both treatments start at the same time. As you move down the list, option B becomes less attractive because you have to wait longer before it starts. In some row, you will probably choose option A.

 S1.3. Example Instructions – Individual Losses

In this section we ask you to imagine that you have the following health state (Health State Z):

 -You have **no** problems in walking about.

-You have **no** problems in washing or dressing yourself.

-You have **no** problems doing your usual activities (e.g. work, study, housework, family or leisure activities).

-You have **no**pain or discomfort.

-You are **not** anxious or depressed.

Unfortunately, you have contracted a disease, for which you will need treatment, at some point in your life to avoid immediate death. If you do not get treatment, you will die in 40 years. In the time between now and the moment of your treatment, this disease will not affect your quality of life. So you could, in principle, live 40 more years without noticing any difference. After treatment (whenever you decide to get treated), you will be able to live out the rest of your life without noticing any problems related to this disease.

**- Treatment A** leads to moderate pain or discomfort and moderate problems with performing your usual activities, for exactly one month (Health State X).

**- Treatment B** will lead to slight pain or discomfort and no problems to perform your usual activities with, for exactly one month (Health State Y).

You are faced with several choice lists about Treatment A and B at different points in time. In the first row, you will probably choose option B, because it offers a smaller loss in health and both treatments start at the same time. As you move down the list, option B becomes more attractive because you can choose to wait longer before it starts. In some row, you will probably choose option A.

 S1.4. Example Instructions – Societal Gains

We now ask you to imagine that a group of 50 students (not including you) has Health State Z (chronic back pain), after contracting a disease. You don’t know any of these students. Health State Z means that:
    · They have **moderate problems** in walking about.

    · They have **no** problems in washing or dressing yourself

    · They have **moderate problems**performing your usual activities.
      (e.g. work, study, housework, family or leisure activities)
    · They have **moderate pain** or discomfort.

    · They are **not** anxious or depressed

In this case there are two treatments that give them a temporary relief of their symptoms:

**-Treatment A** completely takes away the pain during one month. In addition, it allows them to walk with only slight problems and to perform their usual activities with no problems. However, this treatment is expensive and can only be given to 40 of the 50 students. The other 10 students get no treatment.

**-Treatment B** generates the same health effects as Treatment B, but is cheaper and can be given to all 50 students.

The effects of the treatments start immediately at the beginning of the treatment and last for exactly one month. After this the students return to their usual health state Z.

In the first row, you will choose option B, because it offers a larger improvement in health and both treatments start at the same time. As you move down the list, option B becomes less attractive because you have to wait longer before it starts. In some row, you will probably choose option A.

 S1.5. Example Instructions – Societal Losses

We now ask you to imagine that a group of 50 students (not including you) has Health State Z. You don’t know any of these students. Health State Z means that:

 -You have **no** problems in walking about.

-You have **no** problems in washing or dressing yourself.

-You have **no** problems doing your usual activities (e.g. work, study, housework, family or leisure activities).

-You have **no**pain or discomfort.

-You are **not** anxious or depressed.

Unfortunately, the students have contracted a disease, for which they will need treatment, at some point in their life to avoid immediate death. If the students do not get treatment, they will die in 40 years. In the time between now and the moment of their treatment, this disease will not affect their quality of life. So they could, in principle, live 40 more years without noticing any difference. After treatment (whenever they get treated), the students will be able to live out the rest of their life without noticing any problems related to this disease.

There are two treatments available that can completely cure them, but these treatments will result in temporary side effects, with the following symptoms:

**- Treatment A** will lead to slight pain or discomfort but will allow students to perform their usual activities with no problems, for exactly one month. This treatment will have side effects for 40 out of 50 students.
**-Treatment B** has the same side effects as Treatment A, but will result in side effects for all 50 students.

 The effects of the treatments start immediately at the beginning of the treatment and last for exactly one month. After this period the students return to their usual health state Z.

In the first row, you will choose option A, because it leads to side effects for fewer students, and both treatments start at the same time. As you move down the list, option B becomes more attractive because you get to wait longer before it starts. In some row, you will probably choose option B.

 S1.6. Example Choice lists

**S1 Fig. 1. Example of first stage individual choice list, with s = 0**

**S1 Fig. 2. Example of second stage individual choice list, with s = 0, if first stage yields 2 years.**
