# Supplementary figures and images for: Good things come to those who wait—Decreasing impatience for health gains and losses
Source: PLoS One. 2020 Mar 3;15(3):e0229784. doi: 10.1371/journal.pone.0229784 (PMC7053719; doi:10.1371/journal.pone.0229784)

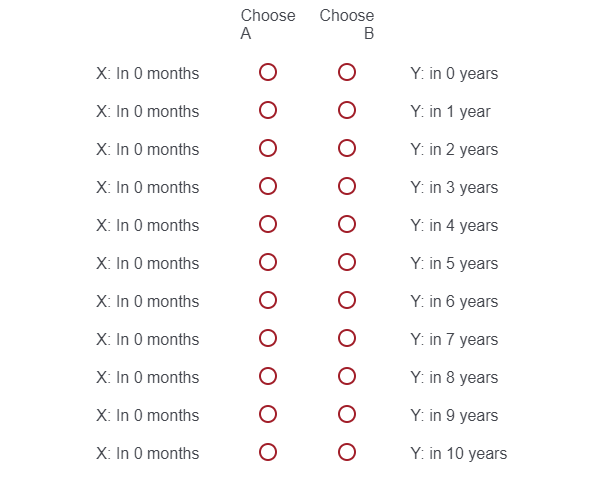

Supplement: S1 Fig — (TIF) [file pone.0229784.s002.tif]

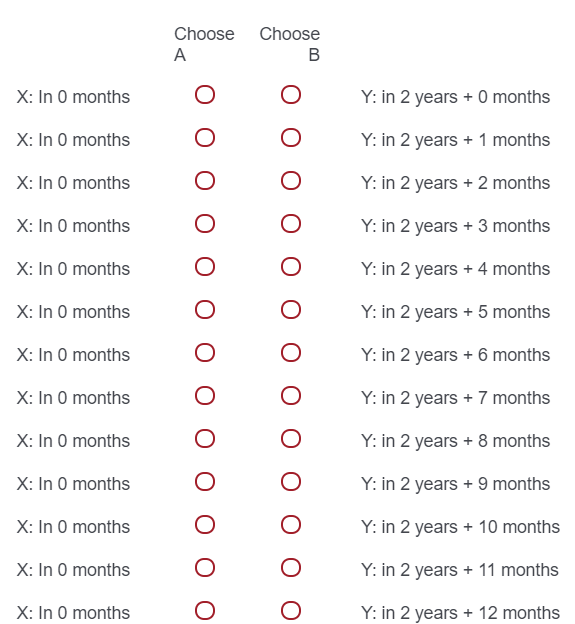

Supplement: S2 Fig — (TIF) [file pone.0229784.s003.tif]
